# Antifoaming Agent for Lubricating Oil: Preparation, Mechanism and Application

**DOI:** 10.3390/molecules28073152

**Published:** 2023-03-31

**Authors:** Chenfei Ren, Xingxing Zhang, Ming Jia, Chenming Ma, Jiaxin Li, Miaomiao Shi, Yunyin Niu

**Affiliations:** Green Catalysis Center, College of Chemistry, Zhengzhou University, Zhengzhou 450001, China

**Keywords:** lubricating oil, silicone-type defoaming agent, non-silicone-type defoaming agent, compound defoaming agent, copolymer, review

## Abstract

In the process of using lubricating oil, it is inevitable that bubbles will be produced, which can not only accelerate the oil’s oxidation and shorten the oil change cycle but also reduce its fluidity and lubricity, aggravate the wear of mechanical parts and produce an air lock that interrupts the oil pump supply and causes an oil shortage accident. This paper mainly and comprehensively discusses the foaming process and its harm, the defoaming mechanism and defoaming method of lubricating oil, more specifically, the synthesis, application, advantages, disadvantages and current situation of three kinds of chemical defoaming agents, namely silicone defoaming agent, non-silicone defoaming agent and compound defoaming agent. Finally, the paper looks forward to the future development of special defoaming agents for lubricating oil.

## 1. Introduction

Lubricating oil is commonly used to reduce friction and wear as well as extend the service life of equipment. Lubricating oil is composed of 90% base oil and a variety of functional additives [1]. Lubricating oil is mainly divided into industrial oil and automobile lubricating oil; automobile lubricating oil is mainly divided into internal combustion engine oil [2], automobile gear oil [3], automobile brake oil, engine oil, water tank and cooling system oil, automatic wave tank oil, grease and so on [4]. When used, due to rapid agitation, impact and injection, lubricating oil will inevitably contact air and produce foam. If the foam cannot be eliminated in time, it will bring a lot of harm to the oil-using equipment and the lubricating oil itself [5]. In addition, in the face of an increasingly harsh working environment, various additives need to be added to the lubricating oil to enhance its performance. Graphene-family materials are used as lubricating additives for various liquids. Graphene-family additives can be dispersed well and stabilized in water-based lubricants due to hydrogen bond interactions [6]. However, for lubricating oils, graphene family additives are generally difficult to disperse and stabilize in oils [7]. Graphene oxide quantum dots (GOQDs) can be used as nano-additives to achieve macro super lubricity. This discovery is conducive to the development of new functional additives for industrial applications [8]. However, the interaction between additives, while improving one property of the oil, may have adverse effects on other properties. For example, cleansing dispersants, antioxidants and anticorrosive agents, and other additives are mostly surfactants that increase the foam formation trend and foam stability of oil products [9]. In running mechanical equipment, foam not only reduces the lubrication effect and aggravates the wear of mechanical parts, but also can produce an air lock to interrupt the oil pump supply and cause an oil shortage accident. As for the lubricating oil itself, the contact area between the foam and the air increases, and operation under high temperature conditions accelerates the oxidation and deterioration of the lubricating oil and shortens the oil change cycle. Therefore, the elimination of harmful foam is of great safety and economic significance for reducing non-essential losses and extending mechanical life. At present, adding antioxidant or defoaming agents (also known as antifoaming agents) into lubricating oil is a relatively simple and effective method [10,11]. Based on this, the action mechanism of lubricating-oil defoaming agents will be discussed in this review, and the types and characteristics of existing defoaming agents will be summarized in order to provide ideas for the research and development of defoaming agents [5].

## 2. Formation and Harm of Foam

### 2.1. Formation of Foam

**Figure 1:**
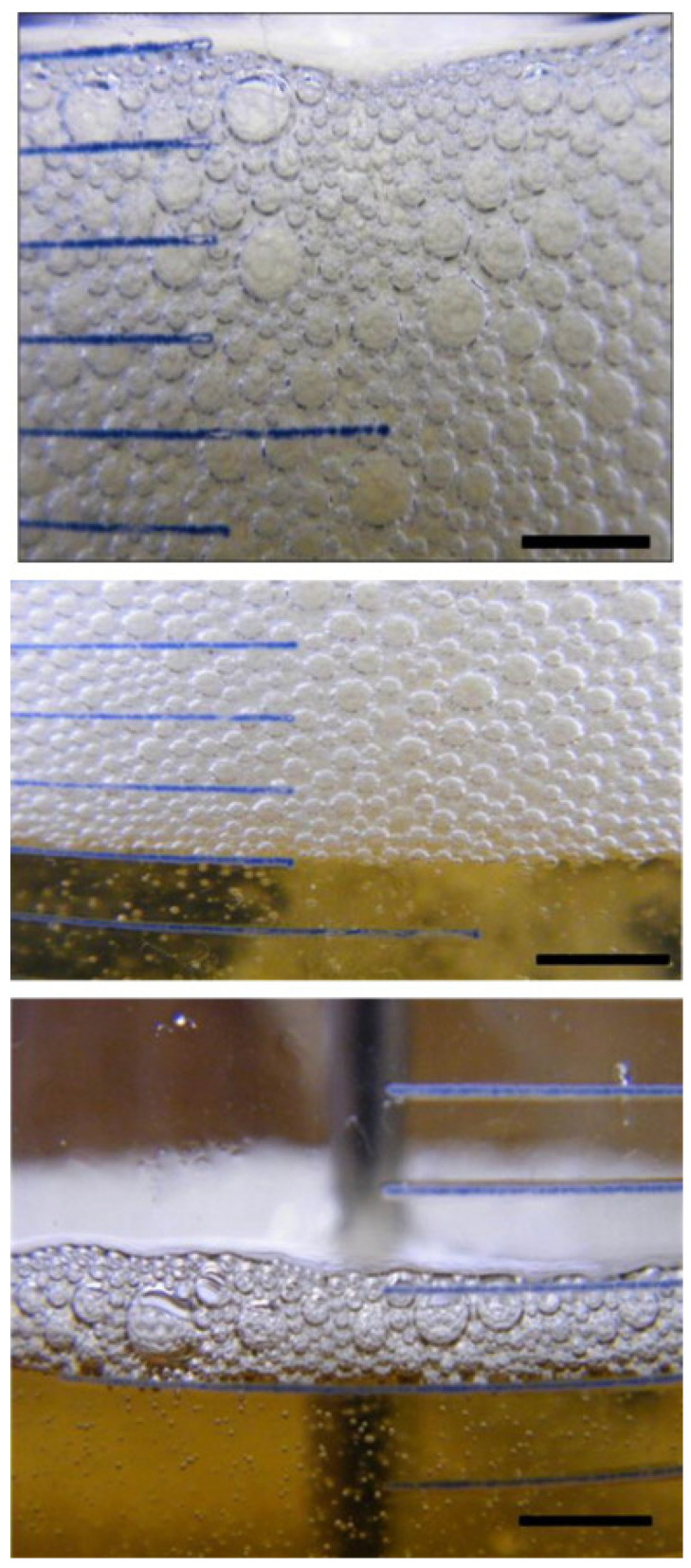
Optical images of the foam produced by a solution of 2 wt.% of additive D in base oil at 20 °C. Reproduced with permission [12]. Copyright © 2010 Elsevier B.V.

Foam is a kind of gas–liquid interfacial phenomenon formed by air and oil, which is a dispersion system with lubricating oil as the dispersion medium and air as the dispersion phase. When bubbles in lubricating oil rise, they are surrounded by a certain thickness of oil film and then form bubble aggregates, as shown in Figure 1. In addition, lubricating oil produces bubbles in contact with air due to rapid stirring in use, as shown in Figure 1 [12].

However, foams are not prone to forming in pure substances, and even frothing will break up and disappear immediately [11]. Therefore, foam that can exist stably in lubricating oil must be caused by the large amount of surfactants in the oil. Foam formation is influenced by the chemical and physical properties of the lubricant as well as by the operating conditions (temperature, pressure, circulating rate of oil in the system, etc.). In some cases, foaming may be caused by additives in the oil formulation [13]. For example, most of cleansing dispersants, antioxidants, anticorrosive agents, coagulants [14] and other additives used in general oils are surfactants. The polar groups of surfactants enriched at the gas–liquid interface point to the liquid, while the non-polar groups point inside the bubbles, forming a single-molecule layer film to reduce interfacial tension, and leaving the bubbles in a more stable thermodynamic state; when the bubbles float up to the liquid surface and escape, the bubble film generates a bimolecular layer film.

### 2.2. Harm of Foam

During the actual use of lubricating oil, due to shock, stirring and other effects, air is mixed into the oil, resulting in the formation of bubbles, which makes the fluidity of lubricating oil worse, the lubricating performance worse, and even produces an air lock that affects the oil supply, so some parts are not lubricated and are worn out or sintered. The harmfulness of foam is as follows:

(1) Degradation of lubrication and wear reduction performance:
  Foam destroys the continuity of the oil film at the friction pair where relative sliding occurs, reduces lubrication performance and causes the parts to lose sufficient lubrication protection, resulting in serious wear and even sintering [9].
(2) Degradation of cooling and heat dissipation performance:
  Partial heat of mechanical equipment can be carried away and dissipated by the lubricating oil when it circulates. However, a large amount of air contained in lubricating oil affects the cooling effect and the heat dissipation effect of the lubricating oil on the machine [15].
(3) Degradation of the cleaning and dispersing effect:
  The contact area between oil and air increases due to foam, and the oxidative metamorphism of lubricating oil at high temperatures intensifies, generating more carbides and sludge; at the same time, lubricating oil with insufficient fluidity cannot adequately flush away the dirty stuffs on the working surface of the parts [9].
(4) Degradation of the anticorrosion and antirust effect:
  Lubricating oil is absorbed on the surface of the parts to form a layer of oil film to isolate oxygen, water, acidic substances and harmful gases in the air to prevent corrosion. Foam not only destroys the oil film but also releases bubbles at high temperatures, creating cavitation [9].
(5) Phenomenon of air lock and flow interruption:
  Because of gas in the oil, on the one hand, the oil produces certain compressibility, which affects pressure transmission; on the other hand, steam resistance is generated, which blocks the oil circuit and affects the oil supply, thus affecting power transmission, making the system unable to work normally, or even interrupting flow and making the lubrication system unable to work normally [16].
(6) Aggravating oxidation and deterioration of lubricating oil:
  When bubbles are generated on the surface or inside the tank, the contact area between the lubricating oil and air increases and, coupled with an increase in oil temperature, aggravates the oxidation and deterioration of the base oil, resulting in a large accumulation of sludge at the bottom of the tank [17].
(7) Potential safety hazard:
  Foam in the lubricating oil increases the volume of the lubricating oil, and lubricating oil may overflow from the oil tank, resulting in oil loss, fire and other unsafe factors [18].

## 3. Defoaming Mechanism

Oil foaming can be divided into two aspects: one is surface foaming, which usually may be controlled by defoaming agents; the other is foaming inside the lubricating oil, which is not easily treated by defoaming agents. Moreover, an effective antifoaming agent on the surface foam may make the inner foam more stable. It is worth noting that different additives have different effects on bubbles in lubricating oil. Some additives will make bubbles aggregate, while others will make bubbles smaller and difficult to remove [11]. In order to obtain a lubricating oil with excellent defoaming properties, special base oil and additives are required to be blended. The defoaming mechanism of a defoaming agent can be divided into the following aspects: (1) the local surface tension of foam decreases, resulting in foam bursting; (2) the defoaming agent can destroy the film’s elasticity and cause the bubble to burst; (3) the defoaming agent can promote liquid film drainage, thus leading to bubble collapse; (4) adding hydrophobic solid particles can lead to bubble collapse; (5) a solubilizing foaming surfactant can lead to bubble bursting [19]. Among the physical properties of lubricating oil, surface tension, density and viscoelasticity of surface film have a great influence on foaming [14]. The keys aspects of the defoaming process are that the (1) surface tension of a defoaming agent is small enough and (2) the insolubility and the highly dispersed colloidal state of a defoaming agent in lubricating oil. Generally, the surface tension of a defoaming agent is in the range of 16–21 mN/m, and that of mineral lubricating oil is in the range of 30–50 mN/m. The greater the difference between the surface tension of the defoaming agent and that of the lubricating oil, the faster the defoaming agent diffuses into the foam film. At the same time, the solubility of a defoaming agent in lubricating oil is required to be small, so that it can have a good defoaming effect with a lower dosage; on the contrary, if the solubility of a defoaming agent in lubricating oil is large, its dissolution in lubricating oil will make the overall surface tension of the lubricant system decrease, thus making bubbles easier to form and difficult to eliminate [20].

## 4. Defoaming Methods

**Figure 2:**
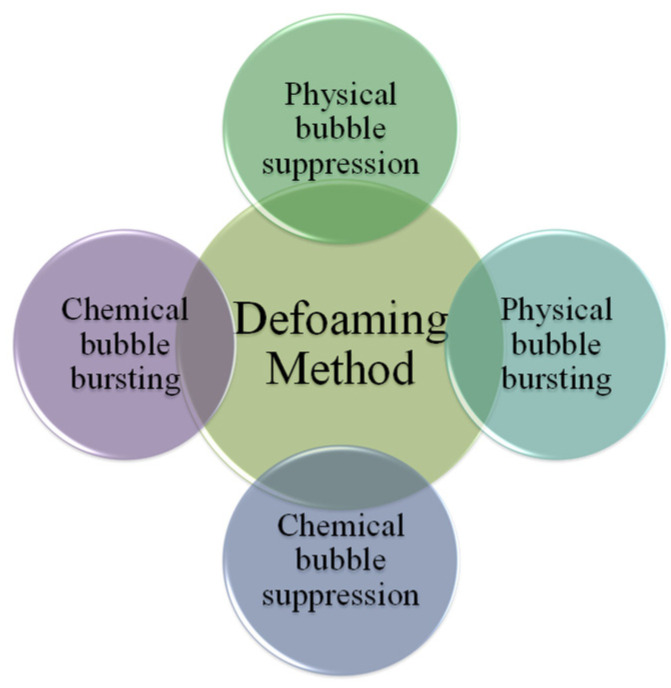
Defoaming methods.

**Figure 3:**
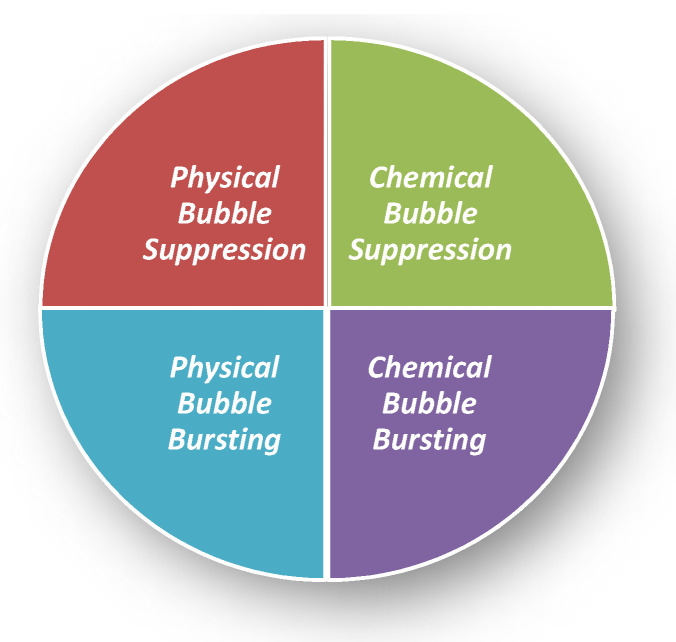
Four kinds of defoaming methods. Physical Bubble Suppression: Temperature change [22]; filtering to remove floating materials [21]; making the vessel open, to remove the mechanical foaming factors (to avoid violent boiling, oscillation, decompression, splashing). Physical Bubble Bursting: Temperature change [22] (freezing, heating [23], evaporation, drying); pressure change [24] (ultrasonic wave [25] and air injection); liquid injection; stirring and tapping with a hydrophobic metal mesh. Chemical Bubble Suppression: Adding defoaming agents; adding defoaming gases; using low foaming surfactants; adding electrolytes; adding substances that eliminate foam stability [21]. Chemical Bubble Bursting: Addition of electrolytes or by electrolysis to weaken the repulsion of the double electrical layers, adding substances discharging liquid [21]; salting out [26].

There are many defoaming methods. Generally, they could be divided into physical defoaming methods and chemical defoaming methods, as shown in Figure 2. However, there are two more implications, i.e., bubble suppression and bubble breaking [21], as shown in Figure 3. To suppress bubbles is to prevent the generation of bubbles, that is, nip in the bud; to destroy bubbles is to eliminate the bubbles that have been created, that is, suit the remedy to the case. Among them, the method of adding defoaming agents belongs to chemical methods.

### 4.1. Physical Defoaming

#### 4.1.1. Physical Bubble Suppression

Temperature change [22]; filtering to remove floating materials [21]; making the vessel open to remove mechanical foaming factors (to avoid violent boiling, oscillation, decompression, splashing); avoiding putting in coarse-faced porous bodies; removing gas phase stirring (only making the liquid phase airtight or covering the liquid surface with a cap); degassing of dissolved stored gas, pre-dehydration of oil by heating, and removal of easily foaming solutes by the method of foaming separation [21] are physical bubble suppression methods.

#### 4.1.2. Physical Bubble Bursting

Temperature change [22] (freezing, heating [23], evaporation, drying); pressure change [24] (ultrasonic wave [25] and air injection); liquid injection; stirring and tapping with a hydrophobic metal mesh; centrifugal separation; radiation; using shallow containers (to disperse bubbles); adding a hydrophobic powder [21] are physical bubble bursting methods.

These physical methods all promote the rate of gas transmission at both ends of the liquid film and the discharge of the bubble film to varying degrees, making the stabilization factor of the foam lower than the decay factor, thus gradually reducing the amount of foam. However, the common disadvantage of these methods is that their use is strongly constrained by environmental factors and the defoaming rate is not high; the advantages are environmental protection and high reusability [27]. 

### 4.2. Chemical Defoaming

#### 4.2.1. Chemical Bubble Suppression

Adding defoaming agents; adding defoaming gases; using low-foaming surfactants; removing foaming substances by using adsorption, precipitation and chemical reactions; adjusting pH [28] and HLB; coating the entire vessel wall with adsorbent agents (to prevent violent boiling); adding substances that increase the solubility of foaming substances; adding electrolytes; adding substances that eliminate foam stability [21] are chemical bubble suppression methods.

#### 4.2.2. Chemical Bubble Bursting

Adding defoaming agents [29]; using adsorption, dissolution, dilution and chemical reaction to remove foaming substances; contacting with volatile gases; adjustment of pH [28] and HLB by the addition of acid and base; removing dispersive bubbles by defoaming agents [23]; addition of electrolytes or by electrolysis to weaken the repulsion of the double electrical layers, adding substances discharging liquid [21]; salting out [26] are chemical bubble bursting methods.

These chemical methods have some shortcomings, such as the uncertainty of foaming substance’s composition, insolubility and harm to system equipment [27]. Nowadays, the most widely used defoaming method is adding a defoaming agent. The biggest advantage of this method lies in high defoaming efficiency and convenient use, but finding a suitable and efficient defoaming agent is the key.

### 4.3. Defoaming Agent

**Figure 4:**
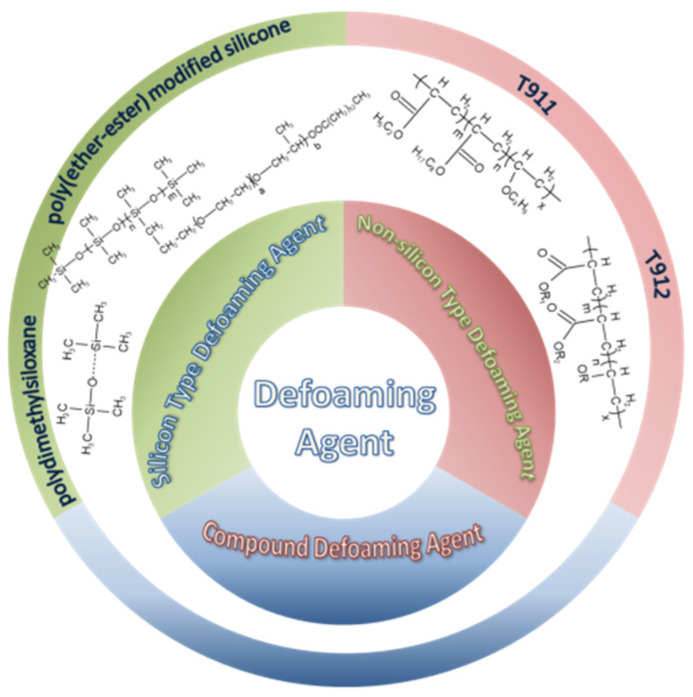
The main types of defoaming agents.

There are many types of defoaming agents: oil type, solution type, emulsion type, powder type and compound type. No matter the type of defoaming agent, in addition to the special requirements of the foaming system, it should have the following properties: (1) strong defoaming power, small dosage; (2) addition to the foaming system does not affect the basic properties of the system; (3) the surface tension is small; (4) good balance with the surface; (5) good diffusivity and permeability; (6) good heat resistance; (7) good chemical stability, strong oxidation resistance; (8) good gas solubility and permeability; (9) small solubility in foaming solution; (10) no physiological activity, high safety [20]. There is no defoaming agent with all of the above conditions at the same time, and a defoaming agent can only be effective for a certain system or several systems. Therefore, in the selection of a defoaming agent, experiments must first be performed. Attention should also be paid to the added concentration. There is a wide variety of defoaming agents, and commonly used defoaming agents are mainly divided into three categories: silicone defoaming agents, non-silicone defoaming agents and compound defoaming agents, as shown in Figure 4.

#### 4.3.1. Silicone-Type Defoaming Agent

**Figure 5:**
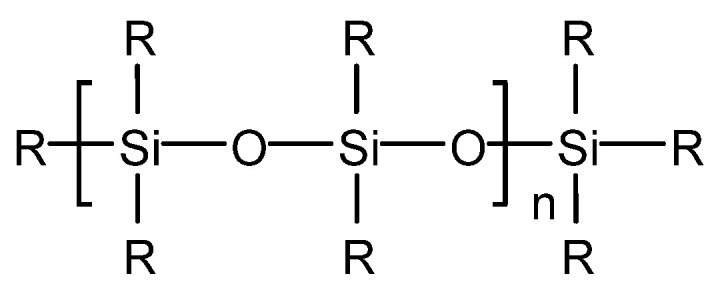
Silicone defoaming agent (R = –CH_3_, the compound is polydimethylsiloxane).

The most common silicone-type defoaming agent is polydimethylsiloxane (T901), commonly known as dimethicone, which is a tasteless, odorless, non-toxic, colorless transparent viscous liquid, a nonpolar organic liquid with a Si-O-Si structure in the main chain [30]. Its structure is shown in Figure 5.

The surface tension of dimethylsilicone oil is low, and the surface tension is between 21 and 25 mN/m at 35 °C. It is a defoaming agent with a good defoaming effect in oil or water. The defoaming effect of silicone-type defoaming agents is greater than the foam inhibition effect. The action mechanism of a silicone-type defoaming agent is that with the aid of insolubility, it distributes in the lubricating oil through a highly dispersed and stable colloid state, which reduces the surface tension of the local lubricating oil film, thus destroying the stability of foam and achieving the effect of eliminating foam. Therefore, a silicone-type defoaming agent has the advantages of strong defoaming ability and lower dosage. Usually, the added amount can have a good defoaming effect between 0.0001% and 0.001%. In addition, a silicone-type defoaming agent has good thermal stability and a good viscosity–temperature property. What is more, the chemical properties of dimethicone in silicone-type defoaming agent are not active, and dimethicone does not easily react with other additives in lubricating oil. At the same time, its small volatilization, high flash point, excellent oxidation resistance and high temperature resistance characteristics make its defoaming effect good. However, the defoaming performance of a silicone-type defoaming agent is related to its dispersion state. For dispersed bubbles, it reduces the surface tension and makes the generated bubbles smaller in diameter and difficult to float, resulting in poor air release. In addition, if a silicone-type defoaming agent is in acid oil, with the passage of time, it will settle due to instability and accumulate, resulting in the failure of defoaming performance. Therefore, the technical requirements of this defoaming agent are more stringent, and different feed ways will also affect the effect and efficiency of defoaming, which needs to be strictly controlled during use. 

In addition, T901 is sensitive to blending technology, is prone to settling and accumulating in lubricating oil and has poor defoaming performance after storage. Zhang Liang et al. [31] have proved through experiments that with the increase of T901 addition, fine particles of the defoaming agent gather into droplets, which damages the surface tension system of the lubricating oil itself, resulting in undesirable phenomena such as the decrease of defoaming performance and the increase of turbidity of the lubricating oil.

Polyether-modified polysiloxane defoaming agent [32] is also a hot research topic in recent years. The main focus is on introducing polyether segments into the polysiloxane chain through block copolymerization or graft copolymerization. The hydrophilic polyether chain segments endow it with water solubility, and the hydrophobic polysiloxane chain segments endow it with low surface tension [33]. This kind of defoaming agent has the advantages of both polyether and silicone defoaming agent. Therefore, it has the characteristics of low surface tension, rapid defoaming, long effective foam suppression time, no toxicity and harm, good stability, low cost, lower dosage, wide application and so on. It is also the most ideal new variety in silicone and has good development prospects.

**Figure 6:**
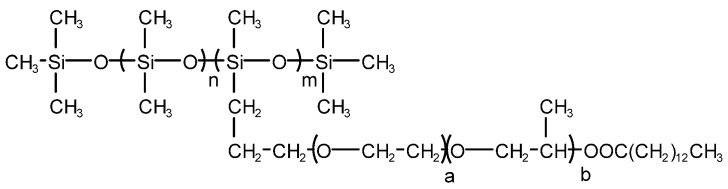
Structure of poly (ether-ester)-modified silicone.

Yinchen Dou et al. synthesized a poly (ether-ester)-modified silicone defoaming agent [34]. The recipe is: hydrophobic silica white, poly (ether-ester)-modified silicone, emulsifier Span80, Tween80, thickening agent glycerol monostearate and water. The defoaming performance of the product was tested, and it was concluded that the surface tension is 28.6 mN/m when the mass concentration of poly (ether-ester)-modified silicone solution is 0.3 g/L. The defoaming time of poly (ether-ester)-modified silicone defoaming agent is 5 s, which is superior to that of a polyether defoaming agent GPE and a silicone defoaming agent X-100F and inferior to that of a silicone defoaming agent SAG. Its foam inhibition height is 300 mL, which is superior to that of a polyether defoaming agent GPE and a silicone defoaming agent X-100F and is equal to that of a silicone defoaming agent SAG. Poly (ether-ester)-modified silicone can rapidly reduce the surface tension at a low mass concentration and has excellent surface performance. Its structure diagram is shown in Figure 6. For the determination of surface tension, the poly (ether-ester)-modified silicone oil was prepared into different concentrations of an aqueous solution at room temperature and measured by a HARKE-A surface tensiometer. For the determination of defoaming performance, the foaming solution was prepared according to GB/T26527-2011 “silicone defoaming agent”, the defoaming performance was determined, and the foaming force and defoaming performance were measured by a cyclic bubbling meter.

**Figure 7:**
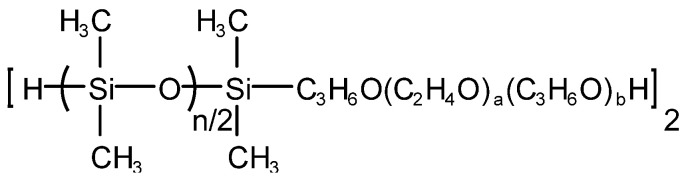
Structure of PESO.

Qiufeng An et al. [35] prepared hydroxyl-capped polyoxypropylene polyoxyethylene oxypropyl-b-polydimethylsiloxane (polyether-b-polysiloxane for short), which is denoted as PESO, and its structure is shown in Figure 7. PESO, dimethyl silicone oil, hydroxyl silicone oil and silicone rubber complex and hydrophobic silica white were added into the three-necked flask equipped with a stirrer, a reflux condenser and a thermometer according to the metering ratio, stirred, heated and warmed up to the set reaction temperature for 30 min. Then, an emulsifier was added and mixed evenly. Finally, deionized water was added while stirring until the solid mass fraction was 45% and a milky white homogeneous liquid could be obtained, namely a nano-effective polyether silicone defoaming agent. The nano-effective polyether silicone defoaming agent has fast foam-bursting speed, relatively long-lasting foam inhibition time, and its performance is close to the level of similar samples.

Yan Hu et al. [36] synthesized a low-silicone defoaming agent with a variety of polyether-modified polysiloxanes as the main body (named polyether-modified polysiloxane defoaming agent) and used a refinery residual oil from CNOOC (China National Offshore Oil Corporation) as the foaming fluid to simulate the delayed coking foaming process in the laboratory. The performance was evaluated by comparing it with that of many different types of delayed coking defoaming agents on the market. The experimental raw materials were: hydrogen-containing silicone oil, allyl polyoxyethylene polyoxypropylene ether (A750, AM7080) and chloroplatinic acid. 

Through the simulated field experiment, 200 mL of residual oil was loaded into the foaming apparatus (the foaming apparatus was assembled by themselves). A micro drug feeder was used to add a quantitative defoamer and then the foaming instrument was sealed. After setting 400 °C and constant temperature for 30 min, the vacuum pump and vacuum valve were opened, vacuuming when the foam height reached 400 mL. The vacuum pump and vacuum valve were closed, and the change of the foam layer with time was monitored. The faster the foam layer is eliminated, the better the performance of the defoaming agent. When the mixed polyether silicone oil defoaming agent was added with a concentration of 20 mg/L, the foam was completely eliminated in 30 s, and it had a low silicone content, which could effectively control the foam in the delayed coking process of residual oil.

#### 4.3.2. Non-Silicone-Type Defoaming Agent

**Figure 8:**
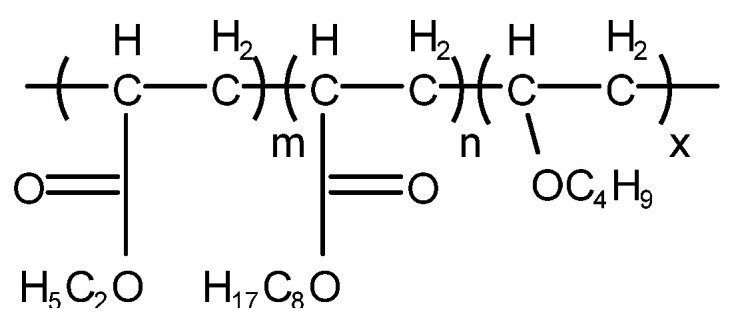
Structure of T911.

**Figure 9:**
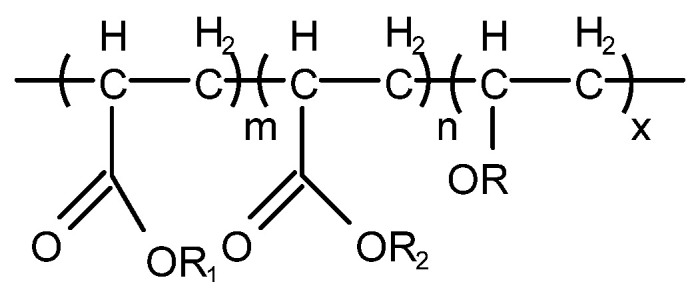
Structure of T912.

Non-silicone-type defoaming agent mainly refers to acrylate or alkyl acrylate copolymer, i.e., acrylate copolymer with different structure, or copolymer of acrylate (or methacrylate) with an ether or ester compound containing a double bond. For some lubricating oils such as turbine oil and hydraulic oil, because long-term use of a silicone defoaming agent leads to the loss of defoaming performance, a non-silicone defoaming agent is usually used. However, a non-silicone defoaming agent has better solubility in mineral oil, and its effect of eliminating foam is not as good as that of a silicone defoaming agent, and the amount added is more than that of a silicone defoaming agent. Among them, the most widely used is polyacrylate [37] (T911 and T912), which is a non-toxic, colorless or slightly yellow transparent viscous liquid. Its structure is shown in Figure 8 and Figure 9.

Polyacrylate has good solubility in mineral oil and has the advantages of low dosage (0.005% to 0.1%), large diameter of generated bubbles, easy release, low impact on air release, insensitivity to various blending techniques, high defoaming efficiency and good defoaming durability in acidic media [38]. Since the bubble suppression effect of a non-silicone defoaming agent is greater than its foam elimination, it is inferred that the action mechanism of a non-silicone defoaming agent is to increase the surface tension between lubricating oil and air, which changes the original system’s tendency of low surface tension and easy-to-form foam. Because of surface activity, it is decided that a non-silicone-type defoaming agent can only properly increase the surface tension of the liquid interface in the system containing surfactants. Therefore, the defoaming property of a non-silicone-type defoaming agent is influenced by the existing surfactant in the system. Wenxuan Huang [39] conducted a test of non-silicone defoaming agent T912 and silicone oil in 250SN base oil with commonly used additives and found that the defoaming performance of T912 deteriorates when used in combination with three additives, T601 (polyvinyl n-butyl ether), T109 (calcium alkyl salicylate) and T705 (barium dinonyl naphthalene sulfonate), and foaming ability was even enhanced. These results indicate that the poor compatibility between a non-silicone defoaming agent and some additives will cause a decline in the defoaming effect and even promote foam generation. Therefore, more attention should be paid to the use of non-silicone defoaming agents. 

**Figure 10:**
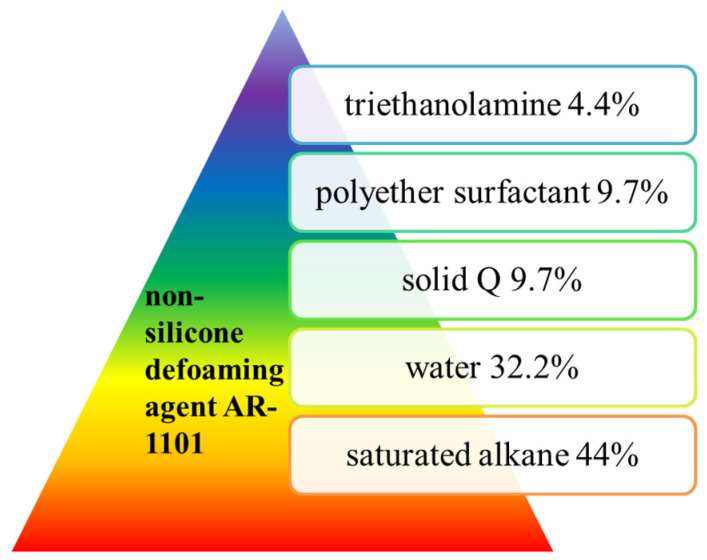
Raw materials for the preparation of a non-silicone defoaming agent AR-1101.

Hong Zhou et al. [40] developed a new non-silicone defoaming agent AR-1101 that has an effect similar to that of an NF defoaming agent. The formula was: polyether surfactant 9.7%, saturated alkane 44%, triethanolamine 4.4%, solid Q 9.7% and water 32.2%, as shown in Figure 10. The above materials were heated and stirred, and when the temperature reached about 90 °C, the oil/water phase emulsion was obtained by holding for several hours. AR-1101 has the appearance of a creamy white thick liquid, non-ionic, with a solid content of more than 40%, pH 6–8, and is non-corrosive. AR-1101, a new multi-component non-silicone defoaming agent, has good stability and a long-lasting defoaming effect. At 50 °C, its defoaming height up to 25 mm, and it is widely used. Although it was popularized in 1987, the critical defoaming temperature needs to be further increased. The full-type measuring cylinder method (AR-1101 and NF comparison test) was used.

**Figure 11:**
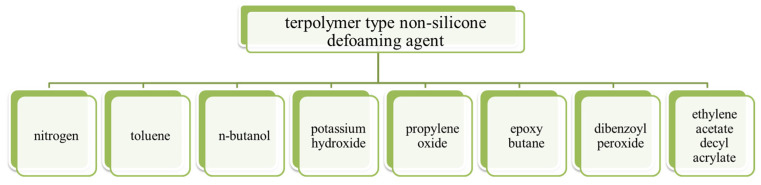
Raw materials for the preparation of a terpolymer-type non-silicone defoaming agent.

Jing Xiong et al. [41] invented a terpolymer-type non-silicone defoaming agent whose formula was nitrogen, toluene, n-butanol, potassium hydroxide, propylene oxide, epoxy butane, dibenzoyl peroxide and ethylene acetate decyl acrylate, as shown in Figure 11. The patent is characterized by the copolymerization of three monomers, including acrylate, ethylene acetate and an epoxy compound. The three monomers have mass percentages of: acrylate 40–60%, ethylene acetate 10–30% and epoxy compound 20–40%; the epoxy compound is a mixture of propylene oxide and epoxy butane. The terpolymer-type non-silicone defoaming agent prepared using this method can be uniformly dispersed in lubricating oil and effectively inhibit the tendency of foam generation. The copolymer is applicable to the field of lubricating oil, and the blended product has efficient defoaming performance and long-term stable performance, which can meet the actual use requirements of the oil. The defoaming performance was carried out by GB/T12579-2002 “determination of foaming characteristics of lubricating oils”.

**Figure 12:**
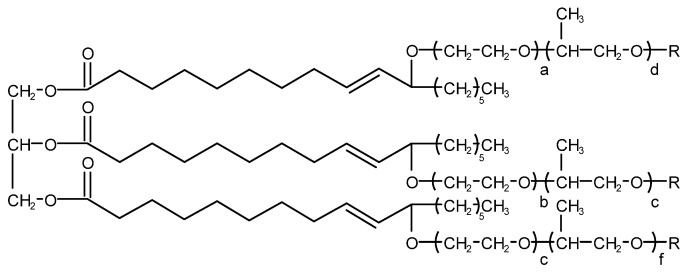
Structure of castor oil polyoxyethylene polyoxylactone oleate (R is selected from H, –OC(CH_2_)_7_CH=CH(CH_2_)_7_CH_3_ at least one R group is –OC(CH_2_)_7_CH=CH(CH_2_)_7_CH_3_, a + b + c = 0–40 and d + e + f = 0–20.).

Yujuan Chen et al. [42] invented a non-silicone defoaming agent and its preparation method. Vegetable oil and its derivatives were used as the supporter, and silica and fatty acid metal soap were used as the main defoaming substances. Because the fatty acid metal soap cannot be swollen in vegetable oil and its derivatives, it exists in the form of particles; although it has good defoaming performance, it can cause the delamination of the defoaming agent. In order to solve this problem, the invention introduces a hydrogenated castor oil substance. On the one hand, through the special process, defoaming performance is ensured, and at the same time, the stability of the product is guaranteed. Through the special process and the secondary introduction of hydrogenated castor oil, the defoaming performance of the product is further improved. Moreover, the introduction of castor oil polyoxyethylene polyoxylactone oleate with a special structure (shown in Figure 12) ensures good compatibility of the product.

Yu Wu et al. synthesized a high-efficiency 2-EHA/VAC copolymer defoaming agent for lubricating oil [43]. Their team used isooctyl acrylate (2-EHA) and vinyl acetate (VAC) as monomers and benzoyl peroxide (BPO) as initiators to synthesize a polymeric non-silicone defoaming agent by dropping mixed monomers into a toluene solvent. Using 2-EHA and VAC as raw materials, the optimum synthesis conditions of 2-EHA/VAC copolymer defoaming agent in toluene solvent were as follows: n(2-EHA):n(VAC) = 0.52:0.48, ω(BPO) = 0.2%, the polymerization temperature was controlled between 80 and 85 °C, the reaction time was 6 h. Its foam stability was 0 mL. The foam resistance of the polymerization product was the best. The surface tension was measured by a BZY-1 automatic surface tensiometer. The process of foam performance measurement was to heat the running oil to 50 °C, add 0.05% of the polyester defoaming agent under mechanical stirring, stir for 10 min and then determine the foam performance at 40 °C according to GB/T12579-2002 “determination of foaming characteristics of lubricating oils”.

#### 4.3.3. Compound Defoaming Agent

Silicone-type defoaming agents and non-silicone-type defoaming agents have their own advantages and limitations [44]. In some cases, the use of silicone-type defoaming agents or non-silicone-type defoaming agents alone cannot meet the defoaming requirements of lubricating oil in terms of air release, defoaming, blending technology, applicable media conditions and so on. For example, there are differences between internal combustion engine oil and gear oil, and because base oil and additives used by different parties are different, the foaming situation of the oil is also different. If only silicone-type agent or non-silicone-type defoaming agents are used, the defoaming effect may not reach the expected result. For hydraulic oil, turbine oil, etc., the refining depth of some oil is not enough, or a variety of additives are added, which makes the effect of a single defoaming agent not good enough. Therefore, in response to these situations, the two types of defoaming agent are compounded according to the appropriate proportions and process. Their strengths and weaknesses are balanced and, combined with the actual situation of the lubricating oil and scientific proportioning, a composite defoaming agent can be developed to achieve a good defoaming effect. Thus, compound defoaming agents were created. At present, the compound defoaming agents that have been widely used are No. 1 compound defoaming agent (T921), No. 2 compound defoaming agent (T922) and No. 3 compound defoaming agent (T923) developed by the Shanghai Refinery Research Institute. T921 is suitable for anti-wear hydraulic oils containing T705 in their formulations and for lubricants with requirements for air release properties. T922 is applicable to diesel engine oils of various brands as well as lubricating oils with high defoaming requirements but no requirements for air release. It has high defoaming performance for lubricating oils containing synthetic sulfonates or other substances with strong foaming properties in the formula [9]. T923 is suitable for medium-speed oil containing a large amount of cleaning dispersants, which has the characteristics of small consumption and a significant defoaming effect, and can be popularized and applied in marine oil [45]. Among them, the No. 3 composite defoaming agent [45] is made of a silicone oil defoaming agent, T912 non-silicone-type defoaming agent, special dispersant and solvent oil agent mixed in the ratio required by the test conditions and stirred well at room temperature. The No. 3 compound defoaming agent was slowly added to 4040 medium-speed engine oil at the temperature range of 70–75 °C under mechanical mixing and then stirred for 10–15 min. After cooling, the foam tendency and foam stability were measured according to GB/T12579-2002 “determination of foaming characteristics of lubricating oils” at 24 °C. At 24 °C, the foaming characteristic (foam tendency/foam stability) of the medium-speed oil without the defoaming agent reached 620/560 mL/mL, with large foaming volume and poor defoaming property. When the No. 2 compound defoaming agent was added at 0.1%, the foaming characteristics were 355/0 mL/mL, and the foaming tendency was still very high. The addition amount of the No. 3 compound defoaming agent was only 0.05%, the foaming characteristic reached 10/0 mL/mL and the defoaming effect was very good (Table 1). The development and use of a variety of compound defoaming agents not only expands the type of defoaming agents, but also makes up for the limitations of silicone and non-silicone defoaming agents.

Yunfang Cao [46] produced a novel 410 compound defoaming agent that was mixed with silicone defoaming agent methylsilicone oil (T901) and non-silicone defoaming agent acrylate ether copolymer (T912). 410 is an effective compound defoaming agent that is suitable for internal combustion engine oil. The recommended dosage is 10–1200 μg/g. When using, the oil is heated to 60 ± 5 °C under mechanical stirring conditions, the 410 compound defoaming agent is added directly and slowly to the oil according to the required amount and stirred evenly. With adding 0.02% of the 410 compound defoaming agent, the foaming characteristic was 10/0 mL/mL, which proved its good defoaming property (Table 1). The application of compound defoaming agent 410 has solved the quality problem of more than 20,000 tons of internal combustion engine oil with unqualified defoaming and obtained obvious economic benefits and certain social benefits. At the same time, considering that the quality change of additives has a complex effect on the bubble resistance of internal combustion engine oil, in order to strengthen the product quality control, it is suggested to carry out the relevant research work on the bubble resistance of internal combustion engine oil and consider the bubble resistance as the quality control index of some additives.

Wei Xu et al. invented a variety of compound defoaming agents for lubricating oil [47], including compound defoaming agents **1**, **2**, **3**, **4** and **5** (Table 1). Among them, compound defoaming agent **1** is prepared by mixing a fluorosilicone oil defoaming agent (FF160), an acrylic copolymer defoaming agent (T912), pentanone and solvent oil for paint (qualified product No. 200 solvent oil) according to the proportions of 1%, 5%, 15%, 79%, at 40 °C with even mechanical stirring. Compound defoaming agent **2** is prepared by mixing fluorosilicone oil (FS1265), a silicone-type defoaming agent (T901), pentanone and aviation kerosene (No. 3 aviation kerosene) in the proportions of 1%, 1%, 20% and 78%, stirring uniformly at 50 °C. Compound defoaming agent **3** is made by mixing fluorosilicone oil (FF160), an acrylic copolymer defoaming agent (T912), pentanone and solvent oil for paint (qualified product No. 200 solvent oil) with proportions of 2%, 8%, 25%, 65%, at 30 °C with even mechanical or artificial stirring. Compound defoaming agent **4** is mixed with fluorosilicone oil (FF160), an acrylate copolymer defoaming agent (T912), a silicone oil defoaming agent (T901), pentanone and solvent oil for paint (qualified product 200 solvent oil) in the ratio of 1%, 3%, 2%, 15%, 79%, prepared by mechanical or manual mixing at a temperature of 20 °C. The compound defoaming agent **5** is prepared by mixing fluorosilicone oil (FF160), a copolymer-type defoaming agent (T912), a silicone oil defoaming agent (T901), pentanone and solvent oil for the paint (qualified No. 200 solvent oil) in the proportions of 1%, 4%, 2%, 16%, and 77%, stirring it evenly with machinery or manually at 50 °C. The evaluation method of high-temperature foam performance of lubricating oil was carried out according to SH/T0722-2002 “standard test method for high temperature foaming characteristics of lubricating oils”. This national standard provides a method for determining the foaming characteristics of lubricating oils (especially transmission fluids and engine oils) at 150 °C. The specific operation method is that the sample is heated, and the inside temperature is kept at a constant level of 49 °C for 30 min, then cooled to room temperature. Then, the sample is transferred to a calibrated 1000 mL measuring cylinder and heated to 150 °C, and dry air is ventilated to the metal diffusion head at the flow rate of 200 mL/min for 5 min. The transient static foam volume before stopping ventilation and after stopping ventilation for 10 min is measured immediately. For lubricating oil, the smaller the amount of static foam and the shorter the time of foam disappearance, the better the high-temperature foaming characteristic of the oil product.

**Table 1:** Defoaming performance parameters of some typical defoaming agents.

Defoaming Agents		Dosage	Foaming Characteristics (Foam Tendency/Foam Stability) (24 °C, mL/mL)	Oil for Test	Data Source
silicone-type defoaming agent	Polydimethylsiloxane	0	650/600	TBN25 marine medium-speed oil	[12] [48] [49] [50]
	(T901)	0.03%	570/470		
Non-silicone-type defoaming agent	Acrylate ether copolymer	0	435/20	Medium extreme-pressure gear oil	[51]
	T911	0.03%~0.1%	0/0		[52]
	Acrylate ether copolymer	0	650/600	TBN25 marine medium-speed oil	[51]
	T912	0.14%	570/280		[50]
	2-EHA/VAC copolymer high-efficiency defoaming agent	0	600/520	Cold heading gear oil	[43]
		0.05%	0/0		
	T921	0	/	Advanced anti-wear hydraulic oil	[52]
		0.005%~0.1%	5/0		
Compound defoaming agent	T922	0	620/560	Shanghai 4040 medium-speed engine oil	[45]
		0.1%	355/0		
	T923	0	620/560	Shanghai 4040 medium-speed engine oil	[45]
		0.05%	10/0		
	410	0	570/530	Diesel engine three-generation oil	[46]
		0.02%	10/0		
	412	0	650/600	TBN25 marine medium-speed oil	[50]
		0.1%	10/0		
	Compound defoaming agent 1	0	240/30 (150 °C)	Internal combustion engine oil	[47]
		0.005%	70/0 (150 °C)	SL5W-30	
	Compound defoaming agent 2	0	210/0 (150 °C)	Internal combustion engine oil	[47]
		0.01%	70/0 (150 °C)	SM5W-30	
	Compound defoaming agent 3	0	210/0 (150 °C)	Internal combustion engine oil	[47]
		0.005%	40/0 (150 °C)	SM5W-30	
	Compound defoaming agent 4	0	210/0 (150 °C)	Internal combustion engine oil	[47]
		0.005%	30/0 (150 °C)	SM5W-30	
	Compound defoaming agent 5	0	190/10 (150 °C)	Continuously variable transmission oil	[47]
		0.005%	50/0 (150 °C)	CVTF	

## 5. Foam Resistance Parameters of Defoaming Agent

Very low surface tension is more conducive to the spread and penetration of defoaming agent molecules on the surface of the foam and accelerates the elimination of foam, so it has stronger defoaming performance. At the same time, the shorter the defoaming time and the higher the defoaming height, the larger the defoaming rate, the closer the foam stability to zero, and the better the defoaming performance of the defoaming agent. Therefore, the defoaming parameters of three kinds of defoaming agents are listed in this paper, as shown in Table 1**.**

## 6. Conclusions and Outlook

Defoaming is the inverse process of bubble stabilization. This paper expounds the mechanism of defoaming agents from two aspects: eliminating foam and inhibiting foam. By far, the most common and effective defoaming agents are silicone-based products, especially polydimethylsiloxane (PDMS) or fluorosilicone products [30]. The application of silicone-based compounds in the separation tank can inhibit the formation of foam but can then cause serious catalyst deactivation in the later stage of the refining process [53]. Therefore, silicone defoaming agents have a good ability to eliminate foam, but the effect of long-term foam inhibition is poor. The initial foam elimination effect of non-silicone defoaming agents is not as good as that of silicone defoaming agents, but their defoaming ability is stable and does not decrease significantly after long-term storage. Compound defoaming agents with good bubble suppression and bubble bursting effects, high dispersibility and good durability will occupy the dominant position in the market to replace single defoaming agents with poor performance and unstable chemical properties [54]. However, the application of compound defoaming agents is a new, and their application scope and methods need to be further studied [55]. The synergistic defoaming effect of the mixture of insoluble hydrophobic particles and hydrophobic oil (filled defoaming agent) dispersed in aqueous media has been well confirmed in the patent literature in the early 1950s. These mixed defoaming agents are very effective at low concentrations (10–1000 ppm) and are widely used [56].

At present, research on the action mechanism of defoaming agents is not clear enough, and there are many related viewpoints, among which the most mainstream accepted theory is the reduction of surface tension at the gas–liquid interface of foam that makes it break. Generally speaking, defoaming agents insoluble in lubricating oil are uniformly distributed in the lubricating oil in the form of fine liquid beads. Because the surface tension of the defoaming agent is smaller than that of the lubricating oil, when the defoaming agent is uniformly distributed in the lubricating oil, it adsorbs on the foam and penetrates into the bubble film, resulting in a change in the strength of the local film and a tension difference. The adsorption film area is subjected to peripheral tension, which causes the film to be gradually thinned and ruptured due to uneven force. On the contrary, if the defoaming agent is dissolved in the lubricating oil, the overall surface tension of the lubricating oil system decreases, and the foam produced by the system is more stable due to the decrease of surface tension.

The use of defoaming agents is the best way to eliminate bubbles in lubricating oil and ensure a normal lubrication system [9]. Therefore, enough attention should be paid to compound defoaming agents, strengthening their research and developing in the direction of lower dosages of defoaming agents, better performance, improved equipment utilization and actual production efficiency. Widely used silicone defoaming agents and non-silicone defoaming agents have their own shortcomings that affect their defoaming effect. Another problem with the industrial use of defoaming agents is “inactivation”, that is, defoaming agents losing their efficiency over time, which means that greater additions are needed. Denkov et al. proposed that so-called “mixing” can lead to the failure of defoaming agents due to the separation of oil and solid components and the emulsification of oil. However, there is little information in the public literature on the inactivation of other types of defoaming agents [57]. Currently, researchers have synthesized polyether-modified silicone oil, which has attracted considerable attention due to its excellent defoaming and foam-inhibiting effects, ease of dispersion and storage stability. With the development of technology, polyether-modified organosilicone-type defoaming agents will gradually replace traditional defoaming agents with single functions and become the mainstream of the future defoaming agent market [10]. In the future, research on polyether-modified polysiloxane defoaming agents can be carried out from the following aspects [58]: (1) Optimizing the structure of polyether-modified polysiloxane from the perspective of molecular design, and preparing polyether-modified polysiloxane with high yield, good performance, strong stability, and environmental protection by adjusting the amount and arrangement formula of ethylene oxide and propylene oxide in the polyether chain segment, the type of polyether end group and the structure of hydrogen-containing silicone oil. (2) Introducing some functional groups to impart other properties to polyether-modified polysiloxanes, suitable for some special foaming systems. (3) For -Si-C- polyether-modified polysiloxanes, seeking low-cost catalysts to reduce production costs; For -Si-O-C- polyether-modified polysiloxanes, seeking suitable additives to reduce the hydrolysis rate of the product and extend the product’s shelf life. (4) Continued exploring of the defoaming mechanism of this type of defoaming agent and optimizing the molecular structure of polyether-modified polysiloxane and composite additives based on the mechanism.

## Data Availability

Not applicable.

## Sample Availability

Samples of the compounds are not available from the authors.
